# Effect of Wnt5a on drug resistance in estrogen receptor-positive breast cancer

**DOI:** 10.1007/s12282-021-01241-0

**Published:** 2021-05-28

**Authors:** Ai Amioka, Takayuki Kadoya, Satoshi Sueoka, Yoshie Kobayashi, Shinsuke Sasada, Akiko Emi, Norio Masumoto, Masaoki Ito, Koh Nakayama, Morihito Okada

**Affiliations:** 1 Department of Surgical Oncology, Research Institute for Radiation Biology and Medicine, Hiroshima University, 1-2-3 Kasumi, Minami-Ku, Hiroshima, 734-8551 Japan; 2 Oxygen Biology Laboratory, Medical Research Institute, Tokyo Medical and Dental University, Bunkyo-ku, Tokyo, 113-8510 Japan

**Keywords:** Wnt5a, Estrogen receptor-positive breast cancer, Cytochrome P450, Pathway analysis, Drug resistance

## Abstract

**Background:**

Previously, we reported that Wnt5a-positive breast cancer can be classified as estrogen receptor (ER)-positive breast cancer; its prognosis is worse than that of Wnt5a-negative breast cancer. This study aimed to investigate the mechanisms underlying the poor prognosis in Wnt5a-positive breast cancer patients.

**Methods:**

In total, 151 consecutive ER-positive breast cancer patients who underwent resection between January 2011 and February 2014 were enrolled. DNA microarray and pathway analyses were conducted using MCF-7 cells stably expressing Wnt5a [MCF-7/Wnt5a (+)]. Based on the outcomes, cell viability/drug sensitivity assays, and mutation analysis were performed using cell cultures and breast cancer tissues. The relationship between Wnt5a and the PI3K–AKT–mTOR signaling pathway was also examined.

**Results:**

The relapse-free survival rate in patients with Wnt5a-positive breast cancer was significantly lower than that in patients with Wnt5a-negative breast cancer (*P* = 0.047). DNA microarray data suggest that only the cytochrome P450 (CYP) pathway was significantly upregulated in MCF-7/Wnt5a (+) cells (*P* = 0.0440). Additionally, MCF-7/Wnt5a (+) cells displayed reduced sensitivity to the metabolic substrates of CYP, tamoxifen (*P* < 0.001), paclitaxel (*P* < 0.001), and cyclophosphamide (*P* < 0.001). Of note, *PIK3CA* mutations were not associated with the expression of Wnt5a in breast cancer tissue and culture cells.

**Conclusions:**

In ER-positive breast cancer, Wnt5a upregulates the CYP metabolic pathway and suppresses tamoxifen, paclitaxel, and cyclophosphamide resistance, all of the three, standard treatment methods for ER-positive breast cancer. Wnt5a is thus potentially involved in the poor prognosis of ER-positive breast cancer independently of the PI3K–AKT–mTOR signaling pathway.

**Supplementary Information:**

The online version contains supplementary material available at 10.1007/s12282-021-01241-0.

## Introduction

The Wnt pathway is classified into the β-catenin-dependent and -independent pathways [1]. Wnt5a is a typical ligand of the β-catenin-independent pathway, modulating cell polarity and migration via the PCP/CE and Ca^2+^ pathways [1, 2]. The Wnt5a signaling pathway is involved in the progression of several cancers [1, 3–7]; Wnt5a contributes to the progression of gastric, lung, and prostate cancers [3–5], but serves as a tumor suppressor in thyroid and ovarian cancers [6, 7]. Wnt5a is reportedly overexpressed in approximately 30% of all breast cancer cases, and most Wnt5a-positive breast cancers are estrogen receptor (ER)-positive breast cancers. Of note, the 5-year relapse-free survival (RFS) rate is significantly lower in Wnt5a-positive versus -negative breast cancer [8].

Ovarian cancer cells overexpressing Wnt5a showed low chemosensitivity to paclitaxel and epirubicin [9]. However, in breast cancers, relatively few studies have examined the association between Wnt5a and drug sensitivity. Furthermore, upregulated PI3K–AKT–mTOR signaling is associated with a poor prognosis in ER-positive breast cancer [10–12]. Of note, Wnt5a is reportedly upregulated in ER-positive breast cancers harboring a *PIK3CA* mutation [13], indicating a close relationship between PI3K signaling and the Wnt5a pathway. Therefore, in the present study, the signaling pathways associated with Wnt5a were investigated to analyze the mechanisms underlying a Wnt5a-mediated drug resistance and poor prognosis.

## Materials and methods

### Patients and breast cancer tissues (RFS-tracked cases)

Consecutive breast cancer tissues resected between 2011 and 2014 were reviewed as previously described [8]. The follow-up period was extended, and the 8-year RFS was investigated by December 2019 in the present study. To strictly investigate “recurrence of early-stage breast cancer,” the two patients with Stage IV at diagnosis included in our previous study [8], were excluded from this study.

Furthermore, we grouped patients treated with the key drugs, tamoxifen, paclitaxel, cyclophosphamide, epirubicin and 5-fluorouracil, and investigated RFS comparing Wnt5a-positive and -negative patients.

### Cell culture

The breast cancer cell lines MCF-7, and MDA-MB-175-VII (RRID: CVCL_0031, and CVCL_1400) were obtained from ATCC (catalog #HTB-22 and #HTB-25, Manassas, VA, USA) and were confirmed not to be listed in the ICLAC Register of Misidentified Cell Lines (version 10). MDA-MB-175-VII is an ER-positive and HER2-negative breast cancer cell line like MCF-7; however, unlike MCF-7, it endogenously expresses Wnt5a [8]. MCF-7 and MDA-MB-175-VII cells were cultured according to the manufacturer’s instructions.

### Transfection, and RNA interference

The pPGK-neo/Wnt5a plasmid was transfected into MCF-7 cells using Lipofectamine LTX + PLUS reagent (Life Technologies, Carlsbad, CA, USA). Successfully transfected cells selectively formed colonies in the presence of G418. The colonies were screened for Wnt5a expression through western blotting. Thereafter, certain MCF-7 cells stably expressing Wnt5a [MCF-7/Wnt5a (+)] or not expressing Wnt5a [MCF-7/Wnt5a (−)] were established. Additionally, the siRNA-mediated suppression of Wnt5a in MCF-7 and MDA-MB-175-VII cells was conducted as previously described [8].

### Gene microarray analyses

The Oligo DNA microarray analyses were performed using 3D-Gene Human Oligo chip 25 k (Toray Industries, Tokyo, Japan) as previously described [8]. In all three MCF-7/Wnt5a (+) clones, only significantly upregulated (expression ≥ twofold) or downregulated (expression ≤ 1/2) genes were selected. Gene ontology and pathway analyses were performed using the DAVID online tool (Version 6.8, https://david.ncifcrf.gov/). In addition, a heatmap was generated using SHINYHEATMAP.COM (http://shinyheatmap.com/). Among the differentially expressed genes, we searched for those associated with breast cancer/cancer/CYP, as per the PubMed website (https://pubmed.ncbi.nlm.nih.gov/). The genes and interrelationships were then arranged using the application “GeneMania” in Cytoscape (Version 3.8.2, http://cytoscape.org).

### Cell viability assay

6 × 10^3^ MCF-7/Wnt5a (+) and MCF-7/Wnt5a (−) cells from ≥ 80% confluent cultures were seeded into a 96-well plate in triplicate. Twenty-four, 48, and 72 h after the administration of 15 µM tamoxifen, 200 nM paclitaxel, 8000 µM cyclophosphamide, 800 nM epirubicin, and 400 µM 5-fluorouracil, 20 µL CellTiter 96^®^ Aqueous One Solution Reagent (Promega, Madison, WI, USA) was added into each well (please see Online Resource 1A). Two hours later, the absorbance was measured according to the manufacture's instruction. Additionally, viability was also measured in the context of MDA-MB-175-VII cells. Briefly, 24 h after seeding (in the same way as for MCF-7 cells) MDA-MB-175-VII were transfected with the Wnt5a-siRNA or negative-siRNA (catalog #4392420 or #4390843, Life Technologies) and viability was measured as above, 24, 48, and 72 h later.

### Western blot analysis

For immunoblot analysis, MCF-7 and MDA-MB-175-VII cells were washed with PBS and lysed with lysis buffer containing a Phosphatase Inhibitor Cocktail (Nacalai Tesque Inc., Kyoto, Japan). Proteins were separated via SDS-PAGE and then electro-transferred onto nitrocellulose membranes (Amersham Protran Premium, GE Healthcare, Buckinghamshire, UK). The membranes were probed with various primary and secondary antibodies (Online Resource 1B) and visualized with enhanced chemiluminescence detection reagents (Amersham ECL Select, GE Healthcare, Buckinghamshire, UK). All western blotting experiments were performed in triplicate.

### Detection of *PIK3CA* mutant variants

Among the 151 cases immunoreactive for Wnt5a, *PIK3CA* mutations were evaluated only in those with a tumor size of ≥ 1 cm in diameter. The QIAamp DNA FFPE Tissue Kit (Qiagen GmbH, Hilden, Germany) was used to extract DNA from formalin-fixed paraffin-embedded (FFPE) tissues. The E542K, E545D/K, and H1047R/L were detected through direct sequencing using the primers listed in Online Resource 1C.

### Quantification of Wnt5a mRNA expression

RNA was extracted using the NucleoSpin total RNA FFPE (Takara Bio, Shiga, Japan) from tissues sections sliced from the FFPE block, including the tumor component only. cDNA was synthesized through reverse-transcription using the PrimeScript II High Fidelity RT-PCR Kit (Takara Bio). Wnt5a expression was quantitatively analyzed via real-time PCR using the SsoFast EvaGreen Supermix (Bio-Rad, Hercules, CA, USA) and the CFX96 real-time PCR detecting system (Bio-Rad). Wnt5a expression was quantified using the ΔCt value. The used primers are listed in Online Resource 1C.

### Statistical analysis

Statistical analysis was performed using the EZR [14] and SPSS (Version 20.0, Chicago, IL, USA) software. Welch's* t* test was used to compare the age, and cell viability between Wnt5a-negative and -positive cells, and Wnt5a-silenced and –non-silenced cells. The clinicopathologic characteristics were analyzed using the Chi-squared test. The significance between RFS curves was analyzed using the generalized Wilcoxon test. The frequency of Wnt5a positivity and the expression levels of Wnt5a mRNA were compared between *PIK3CA* mutation-negative and -positive cases using the Chi-square test and Welch’s *t*-test, respectively. *P* values < 0.05 were considered statistically significant.

## Results

### Wnt5a-positive breast cancer patients show poor prognosis

**Fig. 1:**
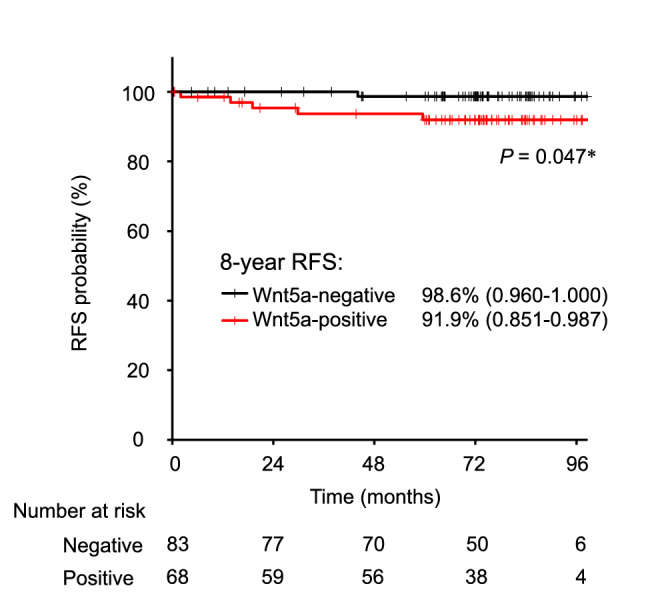
Prognosis of Wnt5a in ER-positive breast cancer patients. Prognosis was estimated via Kaplan–Meier analysis (*n* = 151); Wnt5a-positive breast cancer patients (*n* = 68) displayed a lower 8-year RFS probability: *P = 0.047 (Wilcoxon test). *RFS* relapse-free survival

A total of 151 patients was enrolled. The median (range) follow-up period was 73.2 (11.7 to 102) months for all patients. The background of the patients is shown in Online Resource 2. The 8-year RFS rate of Wnt5a-positive breast cancer patients was lower than that of Wnt5a-negative patients [(91.9% (95% CI = 85.1–98.7) vs 98.6%, (95% CI = 96.0–100.0), *P* = 0.047] (Fig. 1). The postoperative treatment regimens used in recurrent patients are listed in Online Resource 3.

### The CYP pathway is upregulated in Wnt5a-expressing MCF-7 cells

To investigate pathways related to the recurrence of Wnt5a-positive breast cancer, MCF-7 cells stably expressing Wnt5a were established and DNA microarray analyses were performed. A total of 176 candidate genes differentially expressed between MCF-7/Wnt5a (+) and MCF-7/Wnt5a (−) cells, were identified; 76 unique genes were upregulated (expression ≥ twofold) and 100 unique genes were downregulated (expression ≤ 1/twofold; Online Resource 4). The *P*-value was determined using the DAVID database (Table 1). Interestingly, as per the Kyoto Encyclopedia of Genes and Genomes (KEGG) pathway analysis, physiological pathways directly or indirectly influencing Wnt5a expression were identified; only the cytochrome P450 (CYP) pathway, involved in drug metabolism [15], was identified among the upregulated genes (*P* = 0.0440; Table 1). Conversely, certain virus-related pathways were identified among the downregulated genes (Table 1). Interestingly, the JAK-STAT signaling pathway, associated with cancer progression, was attenuated in MCF-7/Wnt5a (+) cells (Table 1). Of note, the upregulated genes were primarily involved in the oxidation–reduction process, response to calcium ions, retinoid metabolic process, response to hypoxia and retinal metabolic process, ATPase binding, and oxidoreductase activity (Table 1). Conversely, most of the downregulated genes include those related to viruses and interferons (Table 1).

**Table 1:** Selected gene categories significantly over-represented in Wnt5a-positive breast cancer

Gene category	Number of genes	%	*P-*value
Upregulated genes			
KEGG pathway			
Drug metabolism—cytochrome P450	3	4.2	0.044
Chemical carcinogenesis	3	4.2	0.059
Gene ontology			
Biological process			
Oxidation–reduction process	8	11	0.006
Response to calcium ion	3	4.2	0.019
Retinoid metabolic process	3	4.2	0.021
Response to hypoxia	4	5.6	0.025
Retinal metabolic process	2	2.8	0.043
Protein polymerization	2	2.8	0.046
Cellular component			
Extracellular exosome	20	28	0.003
Early endosome	5	7	0.009
Plasma membrane	23	32	0.021
Endosome membrane	4	5.6	0.027
Molecular function			
ATPase binding	3	4.2	0.028
Oxidoreductase activity	4	5.6	0.034
Downregulated genes			
KEGG pathway			
Influenza A	13	13.8	< 0.001
Herpes simplex infection	13	13.8	< 0.001
Measles	11	11.7	< 0.001
Hepatitis C	9	9.6	< 0.001
Hepatitis B	6	6.4	0.002
Toxoplasmosis	5	5.3	0.004
Epstein–Barr virus infection	5	5.3	0.006
RIG-I-like receptor signaling pathway	4	4.3	0.008
Chemokine signaling pathway	5	5.3	0.024
JAK–STAT signaling pathway	4	4.3	0.055
Cytosolic DNA-sensing pathway	3	3.2	0.055
Gene ontology			
Biological Process			
Type I interferon signaling pathway	19	20.2	< 0.001
Defense response to virus	22	23.4	< 0.001
Response to virus	19	20.2	< 0.001
Negative regulation of viral genome replication	13	13.8	< 0.001
Interferon-gamma-mediated signaling pathway	10	10.6	< 0.001
Response to interferon-alpha	5	5.3	< 0.001
Innate immune response	12	12.8	< 0.001
Response to interferon-beta	4	4.3	< 0.001
Negative regulation of type I interferon production	4	4.3	< 0.001
Cellular response to interferon-alpha	3	3.2	< 0.001
Positive regulation of interferon-alpha production	3	3.2	0.0015
Positive regulation of interferon-beta production	3	3.2	0.0076
Cellular component			
Cytosol	37	39.4	< 0.001
Cytoplasm	37	39.4	0.0073
Plasma membrane	31	33	0.0076
Spermatoproteasome complex	2	2.1	0.024
Molecular function			
Double-stranded RNA binding	8	8.5	< 0.001
2′-5′-oligoadenylate synthetase activity	4	4.3	< 0.001
NAD + ADP − ribosyltransferase activity	4	4.3	< 0.001
Single-stranded RNA binding	4	4.3	0.001
Nucleotidyltransferase activity	3	3.2	0.0067
Helicase activity	4	4.3	0.0069
Transferase activity	4	4.3	0.0096
CCR5 chemokine receptor binding	2	2.1	0.036

### Relationships between differentially expressed genes, cancer, and CYP

**Fig. 2:**
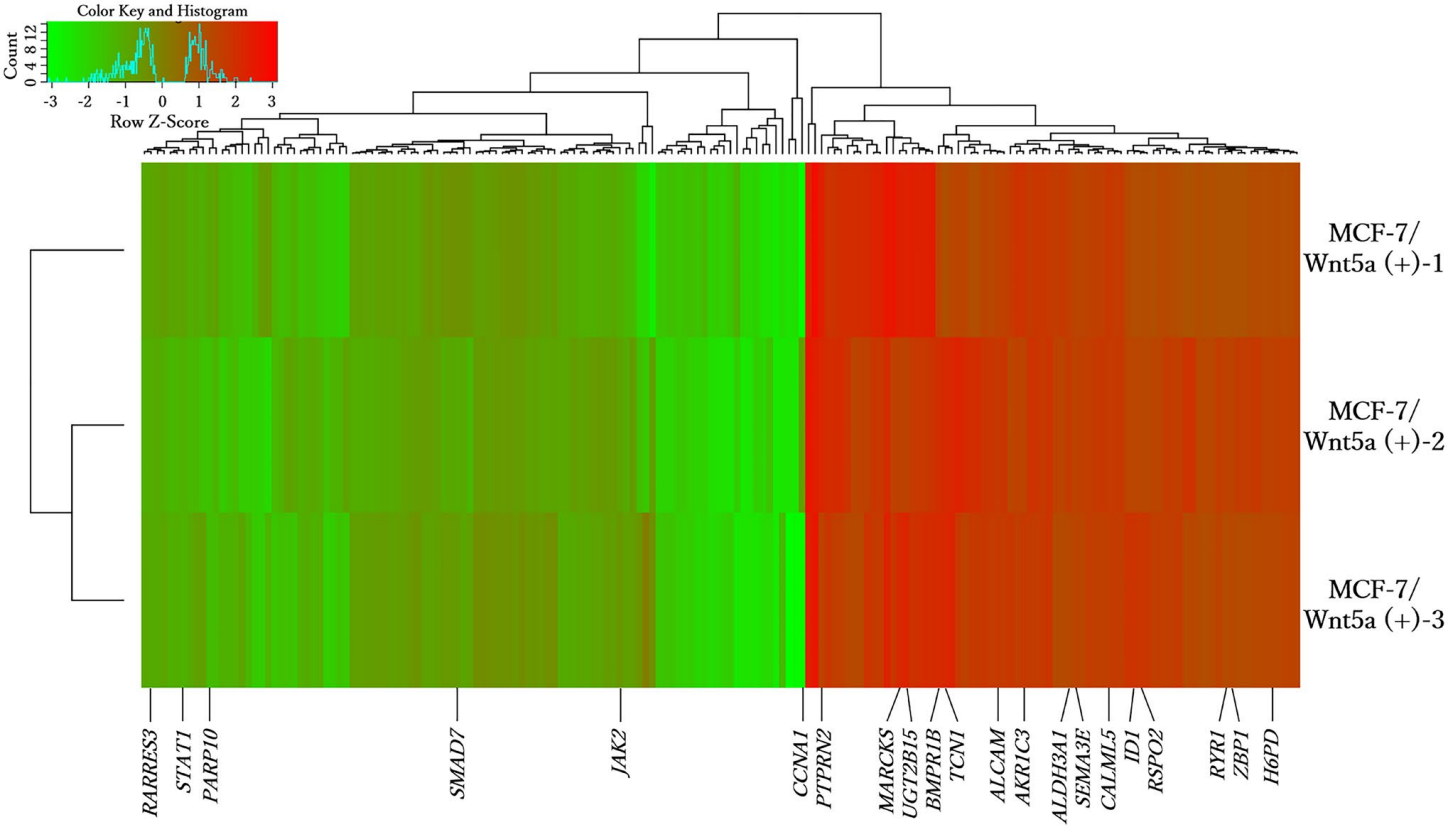
Gene expression heatmap. Gene expression was analyzed via microarray using three MCF-7/Wnt5a (+) clones (MCF-7/Wnt5a (+) 1 to 3; see Online Resource 4). Gene expression was quantified as the relative ratio based on control cells [MCF-7/Wnt5a (−)]. The heatmap was generated at http://shinyheatmap.com/ after the expression levels were logarithmized

The 176 differentially expressed genes are represented in the form of a heatmap (Fig. 2). Among them, 21 genes are related to breast cancer/cancer/CYP and have been reported to promote cell migration and proliferation, and cancer metastasis. The upregulated genes that correlate positively with the malignancy of ER-positive breast cancer are *UGT2B15* [16], *AKR1C3* [17], *ALCAM* [8], and *PTPRN2* [18]; the downregulated genes are *JAK2*, *STAT1* [19], *CCNA1* [20]. Of note, *UGT2B15* and *AKR1C3* are genes related to the CYP pathway, and *ALCAM* and *PTPRN2* are genes co-expressed with *CCNA1* (Online Resource 5).

### Wnt5a expression decreases the sensitivity to tamoxifen, paclitaxel, and cyclophosphamide

**Fig. 3:**
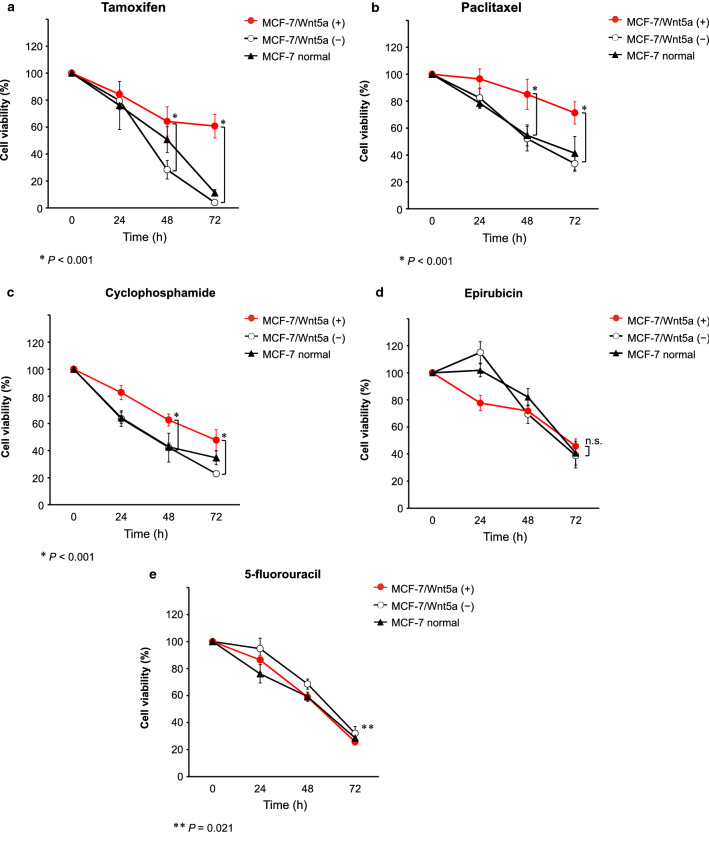
Wnt5a expression diminishes the sensitivity to tamoxifen, paclitaxel, and cyclophosphamide. MCF-7, MCF-7/Wnt5a (+), and MCF-7/Wnt5a (−) cells were exposed to 15 µM tamoxifen (**a**), 200 nM paclitaxel (**b**), 8000 µM cyclophosphamide (**c**), 800 nM epirubicin (**d**), or 400 µM 5-fluorouracil (**e**). Results are represented as the mean ± S.D. of 6 measurements. *P < 0.001; **P = 0.021 (Welch’s *t*-test); *n.s.* not significant

Tamoxifen, paclitaxel and cyclophosphamide, but not epirubicin and 5-fluorouracil, are metabolized by CYP [21–25]. Therefore, the sensitivity to these five drugs, used in the standard treatment of ER-positive breast cancer, was examined. MCF-7/Wnt5a (+) cells exhibited significantly lower sensitivity to tamoxifen, paclitaxel, and cyclophosphamide than MCF-7/Wnt5a (−) cells at both 48 h and 72 h (*P* < 0.001; Fig. 3a–c). In contrast, no difference was found in resistance to epirubicin (*P* = 0.12) and MCF-7/Wnt5a (+) cells exhibited higher sensitivity to 5-fluorouracil (*P* = 0.021; Fig. 3d, e). Importantly, similar trends were observed in the context of MDA-MB-175-VII cells, although without significance in some instances (Online Resource 6).

Additionally, we examined the difference in the RFS between Wnt5a-positive versus -negative breast cancer patients treated with the different above drugs. The RFS probability was lower in the Wnt5a-positive. However, used. However, no significant differences were detected, probably due to the small sample-size (Online Resource 7).

### The PI3K signaling pathway is not correlated with Wnt5a expression

**Fig. 4:**
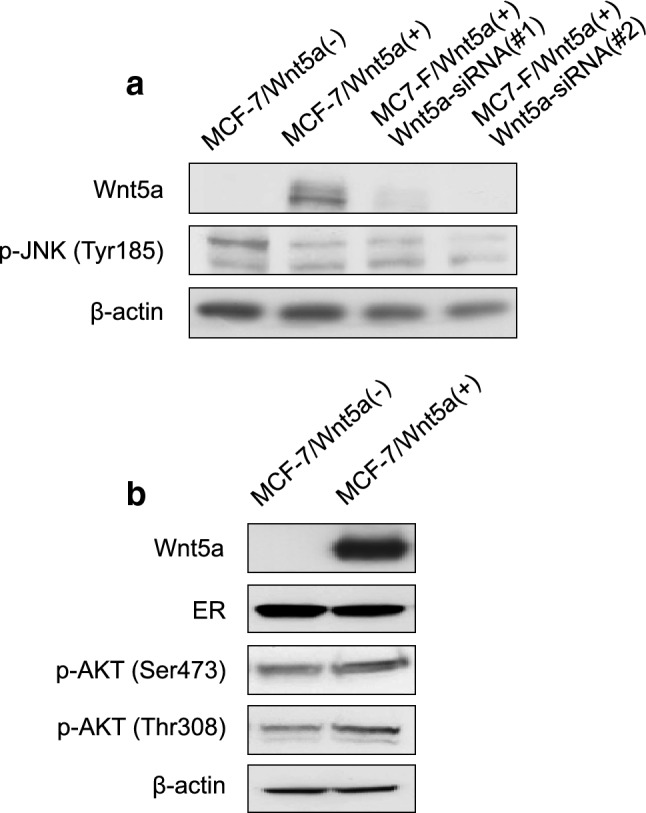
Effect of Wnt5a on the expression of breast cancer-related signaling molecules. The expression of phosphorylated JNK (**a**) and of phosphorylated AKT (**b**) was assessed via western blotting.* ER* estrogen receptor

Next, the relationship between the expression of Wnt5a and the PI3K and JNK signaling pathways was examined via western blotting in MCF-7/Wnt5a (+) and MCF-7/Wnt5a (−) cells. The expression of phosphorylated JNK, which occurs downstream of the Wnt5a signaling pathway [2], remained unaltered in Wnt5a overexpressing or silenced cells (Fig. 4a). Similarly, there was no difference in the expression of phosphorylated AKT (Fig. 4b).

**Fig. 5:**
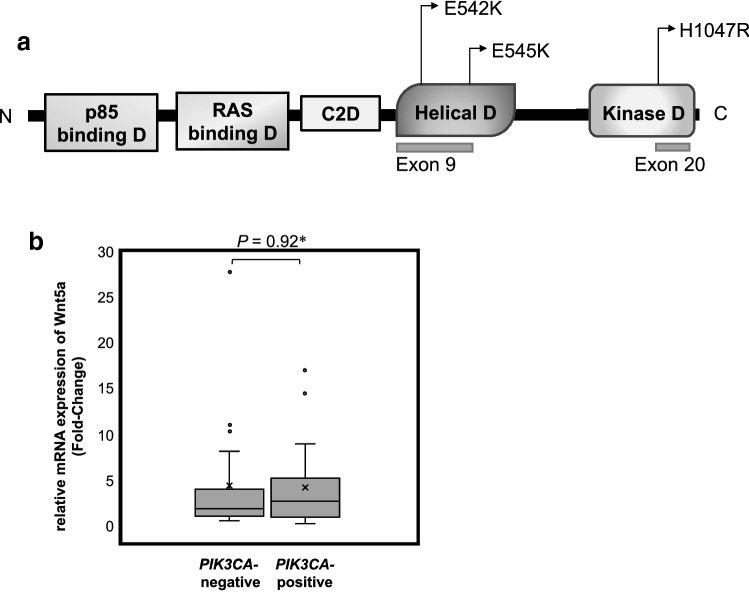
**a** Occurrence of mutations within exons 9 and 20 of *PIK3CA*. **b** Wnt5a mRNA expression according to the *PIK3CA* mutation status (*n* = 40). The Wnt5a mRNA expression was examined in *PIK3CA* mutation-positive and -negative cancers. Statistical significance is highlighted: *P = 0.92 (Welch’s *t*-test)

*PIK3CA* mutations were examined in 40 cases (Table 2) and detected in 19 cases of ER-positive breast cancers (Table 3); three principal mutation sites were identified: E542K, E545K, and H1047R [26] (Fig. 5a). Of note, *PIK3CA* mutations were observed in 8 and 11 Wnt5a-positive and -negative breast cancer patients, respectively. However, there was no significant difference in the frequency of *PIK3CA* mutations depending on the expression of Wnt5a (*P* = 0.73; Table 3). Furthermore, no difference in Wnt5a expression was observed depending on the mutation site (Table 4).

**Table 2:** Characteristics of the 40 ER-positive breast cancer patients assessed for the *PIK3CA* status

	Total (*n* = 40)
Age (median, range)	58.5 (87–34)
≦ 50	15
> 50	25
Tumor size	
pT1 ≦ 20 mm	17
pT2/pT3 > 20 mm	23
Lymph-node metastasis	
Negative	25
Positive	15
Progesterone receptor	
Negative	2
Positive	38
HER2 status	
Negative	37
Positive	3
Nuclear grade	
1, 2	13
3	27
Wnt5a expression (IHC)	
Wnt5a-negative	21
Wnt5a-positive	19

*IHC* immunohistochemistry, *HER2* human epidermal growth factor receptor 2

**Table 3:** Wnt5a expression, assessed through immunohistochemistry (IHC), according to the *PIK3CA* mutation status

Total (*n* = 40)	*PIK3CA* mutation		*P*-value
	Negative (*n* = 21)	Positive (*n* = 19)	
Wnt5a expression (IHC)			
Wnt5a-negative	10	11	
Wnt5a-positive	11	8	0.73

**Table 4:** *PIK3CA* mutation sites in ER-positive breast cancers patients (*n* = 19), as detected through the Sanger method

Wnt5a expression (IHC)	*PIK3CA* mutation			*P-*value
	Exon 9		Exon 20	
	E542K	E545K	H1047R	
Wnt5a-negative	0	6	4	
Wnt5a-positive	1	3	5	0.50

*IHC* immunohistochemistry

Furthermore, the expression of Wnt5a mRNA. The median (range) expression of Wnt5a mRNA was 1.7 (0.94 to 3.9) in *PIK3CA* mutation-negative and 2.5 (0.83–5.1) in *PIK3CA* mutation-positive cases; however, no significant difference was observed between the two groups (*P* = 0.92; Fig. 5b).

## Discussion

The recurrence rate of Wnt5a-positive breast cancer patients is significantly higher than that of Wnt5a-negative breast cancer patients. Therefore, this study investigated the association between the expression of Wnt5a expression and malignancy grade and prognosis. Interestingly, pathway analysis revealed that the CYP metabolic pathway was upregulated after Wnt5a overexpression.

CYP is a key enzyme that oxidizes various substrates and primarily metabolizes drugs in the liver. In our study, CYP upregulation reduced the sensitivity to tamoxifen, paclitaxel, and cyclophosphamide (all metabolized by CYP). Conversely, the sensitivity to epirubicin and 5-fluorouracil (not metabolized by CYP) was not affected. These results suggest that Wnt5a enhances the tamoxifen, paclitaxel, and cyclophosphamide metabolism via CYP, thus decreasing their intracellular concentration. Therefore, the early termination of adjuvant drug therapy for breast cancer may lead to a high recurrence rate of Wnt5a-positive breast cancer. Importantly, as no prior study have shown the association between Wnt5a and CYP in any cancer, these results are novel.

**Fig. 6:**
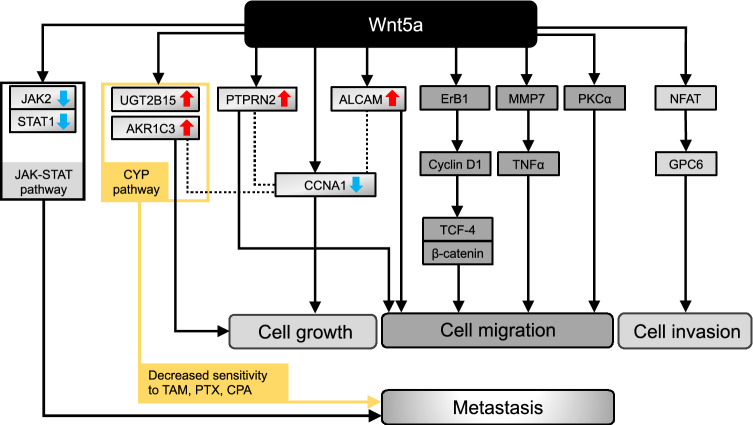
The genes associated with Wnt5a in ER-positive breast cancer are summarized. The solid black lines represent the relationships already reported in the literature; the colored solid lines and the dotted lines and yellow line represent the relationships defined in this study. *TAM* tamoxifen, *PTX* paclitaxel, *CPA* cyclophosphamide

The genes associated in this study with Wnt5a in ER-positive breast cancer are summarized in Fig. 6 together with the previously reported genes [27–30]. Among the upregulated genes, *UGT2B15* and *AKR1C3* are included in the CYP metabolic pathway as per KEGG pathway analyses, suggesting the association between the CYP and Wnt5a pathways in the context of drug sensitivity [16, 17]. Interestingly, *ALCAM* and *PTPRN2* were also shown to correlate with Wnt5a expression and to promote cell migration [8, 18].

Additionally, the downregulation of the JAK-STAT signaling pathway was reported to contribute to breast cancer progression and metastasis [19]. Of note, it has been reported that the downregulation of *CCNA1* leads to cell growth in the context of breast cancer [20]. Interestingly, here we determined that *CCNA1* is co-expressed with *ALCAM*, *PTPRN2*, and *AKR1C3*, suggesting an association between them.

Based on our previous reports [12, 31, 32] we hypothesized that Wnt5a expression would be associated with the PI3K–AKT–mTOR signaling pathway. However, unexpectedly, no significant correlation was found. These results indicate the involvement of pathways other than PI3K in the recurrence of Wnt5a-positive breast cancer.

Altogether, our results indicate that Wnt5a potentially serves as a drug resistance marker in ER-positive breast cancer. However, further studies, in vivo, are essential to validate our results. Additionally, further studies are required for the development of new drugs targeting drug resistance markers; for instance, the development of a treatment strategy for Wnt5a-positive breast cancer based on anti-Wnt5a antibodies, in line with that developed for Wnt5a-positive gastric cancer [33] should be carried out.

In conclusion, here, we show that Wnt5a upregulates CYP expression and diminishes the sensitivity to key drugs used for treating ER-positive breast cancer, including tamoxifen, paclitaxel, and cyclophosphamide. Wnt5a is potentially involved in the poor prognosis of ER-positive breast cancer independently of the PI3K–AKT–mTOR signaling pathway.

## Supplementary Information

Below is the link to the electronic supplementary material.

Supplementary file1 (PDF 124 KB)

Supplementary file2 (PDF 44 KB)

Supplementary file3 (PDF 84 KB)

Supplementary file4 (XLSX 135 KB)

Supplementary file5 (PDF 14374 KB)

Supplementary file6 (PDF 133 KB)

Supplementary file7 (DOCX 137 KB)
