# Supplementary material for: Effect of Wnt5a on drug resistance in estrogen receptor-positive breast cancer
Source: Breast Cancer. 2021 May 28;28(5):1062–71. doi: 10.1007/s12282-021-01241-0 (PMC8354951; doi:10.1007/s12282-021-01241-0)
Supplement: Supplementary file 1 — Supplementary file1 (PDF 124 KB) [file 12282_2021_1241_MOESM1_ESM.pdf]

Effect of Wnt5a on drug resistance in estrogen receptor-positive breast cancer

Ai Amioka[1)], Takayuki Kadoya[1)], Satoshi Sueoka[1)], Yoshie Kobayashi[1)], Shinsuke Sasada[1)], Akiko Emi[1)], Norio Masumoto[1)], Masaoki Ito[1)], Koh Nakayama[2)], Morihito Okada[1)]

1) Department of Surgical Oncology, Research Institute for Radiation Biology and Medicine, Hiroshima University, 1-2-3 Kasumi, Minami-Ku, Hiroshima 734-8551, Japan
2) Oxygen Biology Laboratory, Medical Research Institute, Tokyo Medical and Dental University, Bunkyo-ku, Tokyo 113-8510, Japan

Corresponding author：

Takayuki Kadoya, M.D, Ph.D

Email: takayukikadoya@gmail.com

Tel.: +81-082-257-5869

Fax: +81-082-256-7109

Online Resource 1

A. The materials used in the cell viability assay

Materials	Manufacturing company	catalog number
(Z)-4-hydroxytamoxifen	Sigma-Aldrich (St. Louis, MO, USA)	H7904
Epirubicin (hydrochloride)	Cayman Chemical (Ann Arbor, MI, USA)	1640184
Paclitaxel	FUJIFILM Wako Pure Chemical Corporation (Osaka, Japan)	33069-62-4
Cyclophosphamide	FUJIFILM Wako Pure Chemical Corporation (Osaka, Japan)	030-12953
5-fluorouracil	FUJIFILM Wako Pure Chemical Corporation (Osaka, Japan)	068-01401

B. List of antibodies used in western blot analysis

Antibody	Manufacturing company	catalog number
Anti-Wnt5a/b rabbit monoclonal antibody	Cell Signaling Technology (Beverly, MA, USA)	2530
Anti-phospho-AKT (Ser473) antibody	Cell Signaling Technology (Beverly, MA, USA)	9271
Anti-phospho-AKT (Thr308) rabbit monoclonal antibody	Cell Signaling Technology (Beverly, MA, USA)	4056
Anti-phospho-SAPK/JNK (Tyr185) antibody	Cell Signaling Technology (Beverly, MA, USA)	9251
Anti-ER antibody	Abcam (Cambridge, UK)	ab16660
Anti-HER2 antibody	Abcam (Cambridge, UK)	ab214275
Anti-β-actin antibody	Sigma-Aldrich (St Louis, MO, USA)	A5441

C. The nucleotide sequences of the primers used in this study

Primer	Nucleotide sequence
E542K and E545D/K	5′-GCTAGAGACAATGAATTAAGGGAAAA-3′ (forward) and 5′-CTCCATTTTAGCACTTACCTGTGAC-3′ (reverse)
H1047R/L	5′-GCAAGAGGCTTTGGAGTATTT-3′ (forward) and 5′-GTGTGGAAGATCCAATCCATTT-3′ (reverse)
Wnt5a	5′-AAGTTGGTACAGGTCAACAGCCGCT-3′ (forward) and 5′-CACATGAGCTCGCAGCCATCCATG-3′ (reverse)
β-actin.	5′-TGAGCGCGGCTACAGCTT-3′ (forward) and 5′-TCCTTAATGTCACGCACGATTT-3′ (reverse)
