# Supplementary material for: Effect of Wnt5a on drug resistance in estrogen receptor-positive breast cancer
Source: Breast Cancer. 2021 May 28;28(5):1062–71. doi: 10.1007/s12282-021-01241-0 (PMC8354951; doi:10.1007/s12282-021-01241-0)
Supplement: Supplementary file 2 — Supplementary file2 (PDF 44 KB) [file 12282_2021_1241_MOESM2_ESM.pdf]

Effect of Wnt5a on drug resistance in estrogen receptor-positive breast cancer

Ai Amioka[1)], Takayuki Kadoya[1)], Satoshi Sueoka[1)], Yoshie Kobayashi[1)], Shinsuke Sasada[1)], Akiko Emi[1)], Norio Masumoto[1)], Masaoki Ito[1)], Koh Nakayama[2)], Morihito Okada[1)]

1) Department of Surgical Oncology, Research Institute for Radiation Biology and Medicine, Hiroshima University, 1-2-3 Kasumi, Minami-Ku, Hiroshima 734-8551, Japan
2) Oxygen Biology Laboratory, Medical Research Institute, Tokyo Medical and Dental University, Bunkyo-ku, Tokyo 113-8510, Japan

Corresponding author：

Takayuki Kadoya, M.D, Ph.D

Email: takayukikadoya@gmail.com

Tel.: +81-082-257-5869

Fax: +81-082-256-7109

Online Resource 2

Correlation between the Wnt5a status and the clinical characteristics in ER-positive breast cancer patients

			**Wnt5a (IHC)**		
		**Total (*n*=151)**	**Negative (*n* = 83)**	**Positive (*n* = 68)**	***P*-value**
**Age at surgery, n (%)**					
Median age at surgery (IQR)		61.0 (47-69)	63.0 (47-69)	57.5 (46-68)	0.19
**Tumor size**					
pT1		103 (68)	62 (75)	41 (60)	
pT2/T3		48 (32)	21 (25)	27 (40)	0.059
**Nodal metastasis**					
Negative		103 (68)	72 (87)	31 (46)	
Positive		48 (32)	11 (13)	37 (54)	<0.001
**HER2 status**					
Negative		142 (94)	77 (93)	65 (96)	
Positive		9 (6.0)	6 (7.2)	3 (4.4)	0.47
**Hormonal therapy**					
Yes	TAM	41 (27)	22 (27)	19 (28)	

	AI	83 (55)	46 (55)	37 (54)	
	TAM and AI sequentially	17 (11)	9 (11)	8 (12)	
No		10 (6.6)	6 (7.2)	4 (5.9)	0.74
**Adjuvant chemotherapy**					
Yes	FEC-DTX/PTX	13 (8.6)	2 (2.4)	11 (16)	
	FEC	2 (1.3)	1 (1.2)	1 (1.5)	
	TC	28 (19)	15 (18)	13 (19)	
	Other	2 (1.3)	0 (0)	2 (2.9)	
No		106 (70)	65 (78)	41 (60)	0.016

Abbreviations: TAM, Tamoxifen; AI, Aromatase inhibitor; FEC, 5-Fluorouracil + Epirubicin + Cyclophosphamide; DTX, Docetaxel; PTX, Paclitaxel; TC, Docetaxel + Cyclophosphamide; IQR, interquartile range; IHC, immunohistochemistry
