# Supplementary material for: Effect of Wnt5a on drug resistance in estrogen receptor-positive breast cancer
Source: Breast Cancer. 2021 May 28;28(5):1062–71. doi: 10.1007/s12282-021-01241-0 (PMC8354951; doi:10.1007/s12282-021-01241-0)
Supplement: Supplementary file 3 — Supplementary file3 (PDF 84 KB) [file 12282_2021_1241_MOESM3_ESM.pdf]

Effect of Wnt5a on drug resistance in estrogen receptor-positive breast cancer

Ai Amioka[1)], Takayuki Kadoya[1)], Satoshi Sueoka[1)], Yoshie Kobayashi[1)], Shinsuke Sasada[1)], Akiko Emi[1)], Norio Masumoto[1)], Masaoki Ito[1)], Koh Nakayama[2)], Morihito Okada[1)]

1) Department of Surgical Oncology, Research Institute for Radiation Biology and Medicine, Hiroshima University, 1-2-3 Kasumi, Minami-Ku, Hiroshima 734-8551, Japan
2) Oxygen Biology Laboratory, Medical Research Institute, Tokyo Medical and Dental University, Bunkyo-ku, Tokyo 113-8510, Japan

Corresponding author：

Takayuki Kadoya, M.D, Ph.D

Email: takayukikadoya@gmail.com

Tel.: +81-082-257-5869

Fax: +81-082-256-7109

Online Resource 3

Postoperative treatment of recurrent cases of ER-positive breast cancer

Cases	Wnt5a expression	Adjuvant chemotherapy	Adjuvant hormone therapy
1	positive	−	TAM
2	positive	−	TAM, LH-RHa
3	positive	−	AI
4	positive	TC	AI
5	positive	FEC+DTX	−
6	negative	TC	AI

Abbreviations: TAM, Tamoxifen; LH-RHa, Luteinizing hormone-releasing hormone agonist; AI, Aromatase inhibitor; TC, Docetaxel + Cyclophosphamide; FEC, 5-Fluorouracil + Epirubicin + Cyclophosphamide; DTX, Docetaxel.
