# Supplementary material for: Effect of Wnt5a on drug resistance in estrogen receptor-positive breast cancer
Source: Breast Cancer. 2021 May 28;28(5):1062–71. doi: 10.1007/s12282-021-01241-0 (PMC8354951; doi:10.1007/s12282-021-01241-0)
Supplement: Supplementary file 5 — Supplementary file5 (PDF 14374 KB) [file 12282_2021_1241_MOESM5_ESM.pdf]

Effect of Wnt5a on drug resistance in estrogen receptor-positive breast cancer

Ai Amioka[1)], Takayuki Kadoya[1)], Satoshi Sueoka[1)], Yoshie Kobayashi[1)], Shinsuke Sasada[1)], Akiko Emi[1)], Norio Masumoto[1)], Masaoki Ito[1)], Koh Nakayama[2)], Morihito Okada[1)]

1) Department of Surgical Oncology, Research Institute for Radiation Biology and Medicine, Hiroshima University, 1-2-3 Kasumi, Minami-Ku, Hiroshima 734-8551, Japan
2) Oxygen Biology Laboratory, Medical Research Institute, Tokyo Medical and Dental University, Bunkyo-ku, Tokyo 113-8510, Japan

Corresponding author：

Takayuki Kadoya, M.D, Ph.D

Email: takayukikadoya@gmail.com

Tel.: +81-082-257-5869

Fax: +81-082-256-7109

Online Resource 5

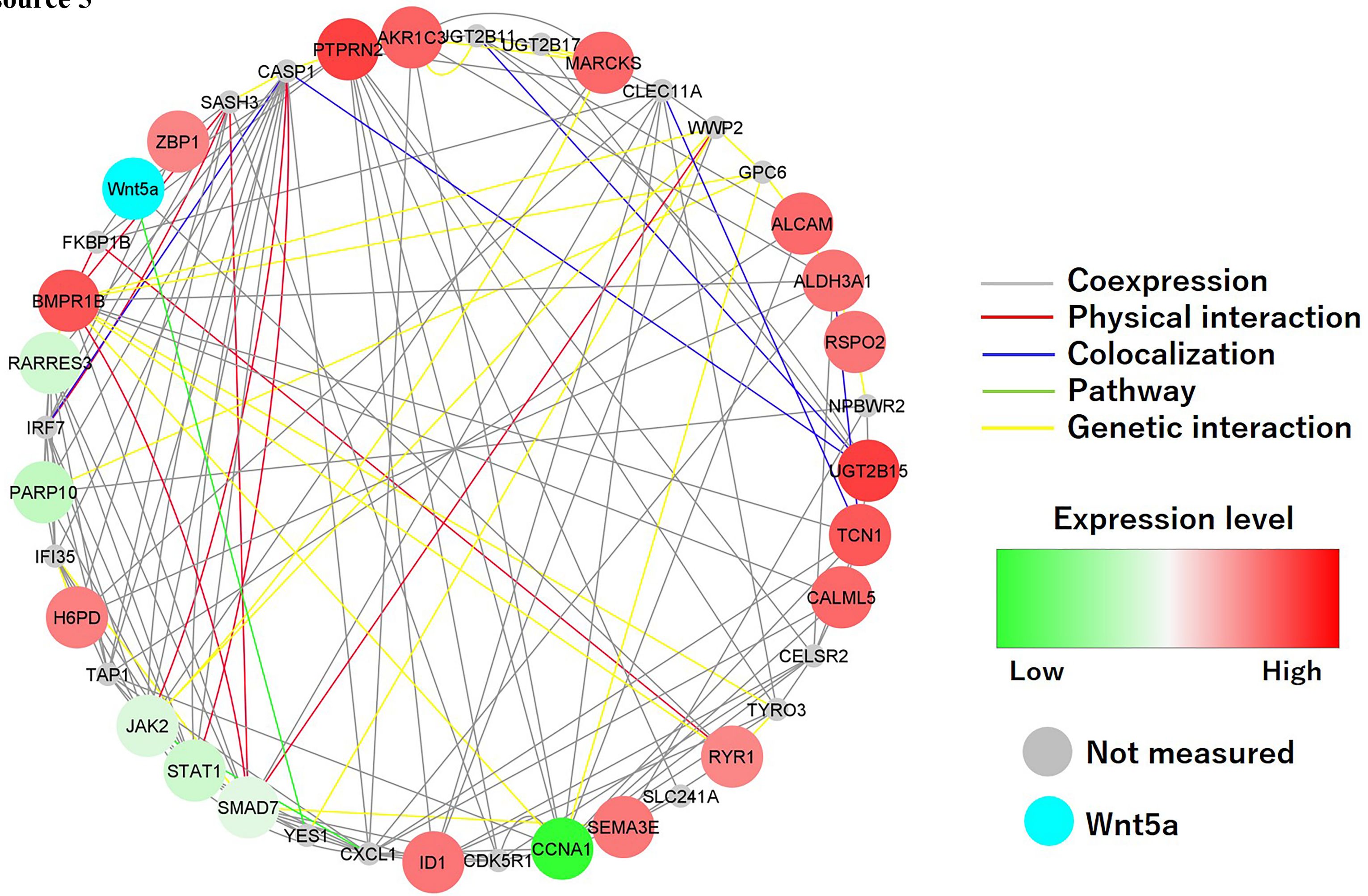

Interrelationship and expression levels of the genes shown in Online Resource 4

Genes were arranged in a circle and their interrelationships were characterized with colored lines using CytoScape (Version 3.8.2, http://cytoscape.org). The gene expression levels after conversion to logarithm are color-coded; from green to red for low or highly expressed genes, respectively. Some genes whose expression was not estimated in DNA microarray analysis are shown as gray circles. The colored lines represent the interrelationships between the connected genes.
