# Supplementary material for: Effect of Wnt5a on drug resistance in estrogen receptor-positive breast cancer
Source: Breast Cancer. 2021 May 28;28(5):1062–71. doi: 10.1007/s12282-021-01241-0 (PMC8354951; doi:10.1007/s12282-021-01241-0)
Supplement: Supplementary file 6 — Supplementary file6 (PDF 133 KB) [file 12282_2021_1241_MOESM6_ESM.pdf]

Effect of Wnt5a on drug resistance in estrogen receptor-positive breast cancer

Ai Amioka[1)], Takayuki Kadoya[1)], Satoshi Sueoka[1)], Yoshie Kobayashi[1)], Shinsuke Sasada[1)], Akiko Emi[1)], Norio Masumoto[1)], Masaoki Ito[1)], Koh Nakayama[2)], Morihito Okada[1)]

1) Department of Surgical Oncology, Research Institute for Radiation Biology and Medicine, Hiroshima University, 1-2-3 Kasumi, Minami-Ku, Hiroshima 734-8551, Japan
2) Oxygen Biology Laboratory, Medical Research Institute, Tokyo Medical and Dental University, Bunkyo-ku, Tokyo 113-8510, Japan

Corresponding author：

Takayuki Kadoya, M.D, Ph.D

Email: takayukikadoya@gmail.com

Tel.: +81-082-257-5869

Fax: +81-082-256-7109

Online Resource 6

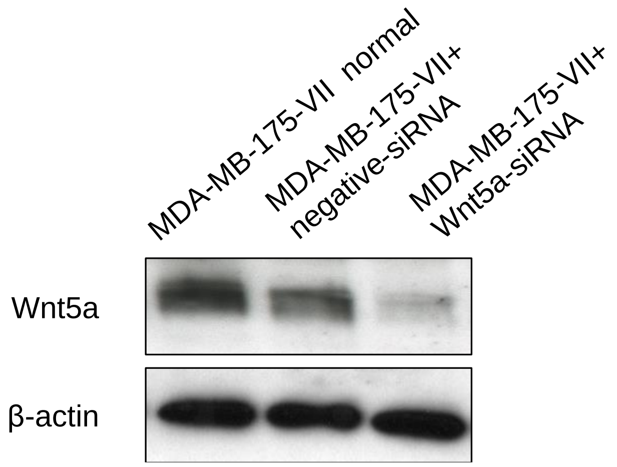

siRNA transfection of MDA-MB-175-VII cells

MDA-MB-175-VII is a breast cancer cell line ER-positive and HER2-negative, like MCF-7 cells; however, unlike MCF-7 cells, MDA-MB-175-VII cells endogenously express Wnt5a. Twenty-four hours after Wnt5a-siRNA transfection, western blot was performed to confirm that Wnt5a was knocked down.

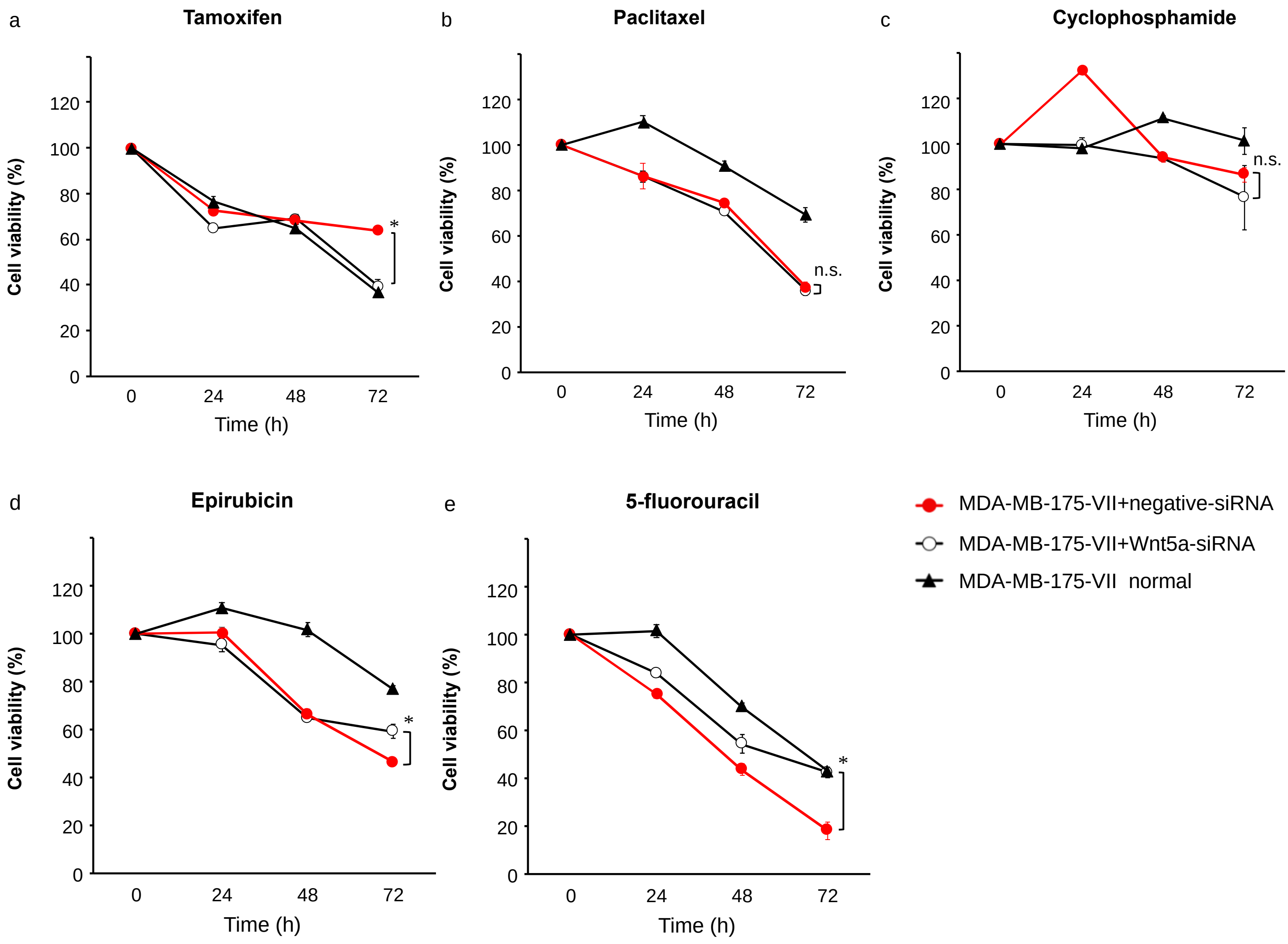

The expression of Wnt5a diminishes the sensitivity to tamoxifen

MDA-MB-175-VII cells were exposed to 5 μM tamoxifen (a), 200 nM paclitaxel (b), 200 μM cyclophosphamide (c), 100 nM epirubicin (d), and 400 μM 5-fluorouracil (e). Data are represented as the mean ± S.D. of 6 measurements. * $P < 0.05$; n.s., not significant.
