# Supplementary material for: Effect of Wnt5a on drug resistance in estrogen receptor-positive breast cancer
Source: Breast Cancer. 2021 May 28;28(5):1062–71. doi: 10.1007/s12282-021-01241-0 (PMC8354951; doi:10.1007/s12282-021-01241-0)
Supplement: Supplementary file 7 — Supplementary file7 (DOCX 137 KB) [file 12282_2021_1241_MOESM7_ESM.docx]

**Effect of Wnt5a on drug resistance in estrogen receptor-positive breast cancer**

Ai Amioka^1)^, Takayuki Kadoya^1)^, Satoshi Sueoka^1)^, Yoshie Kobayashi^1)^, Shinsuke Sasada^1)^, Akiko Emi^1)^, Norio Masumoto^1)^, Masaoki Ito^1)^, Koh Nakayama^2)^, Morihito Okada^1)^

1. Department of Surgical Oncology, Research Institute for Radiation Biology and Medicine, Hiroshima University, 1-2-3 Kasumi, Minami-Ku, Hiroshima 734-8551, Japan
2. Oxygen Biology Laboratory, Medical Research Institute, Tokyo Medical and Dental University, Bunkyo-ku, Tokyo 113-8510, Japan

**Corresponding author：**

Takayuki Kadoya, M.D, Ph.D

**Email:** [takayukikadoya@gmail.com](mailto:takayukikadoya@gmail.com)

**Tel.:** +81-082-257-5869

**Fax:** +81-082-256-7109

**Online Resource 7**

**RFS in Wnt5a-positive *versus* -negative breast cancer patients treated with tamoxifen, paclitaxel, cyclophosphamide, epirubicin, and 5-fluorouracil**

The RFS probability was lower in the Wnt5a-positive patients, irrespectively of the drug used. However, No significant differences were detected, probably because of the small number of cases.
